# Supplementary material for: The psychological impacts of the COVID-19 pandemic on business leadership
Source: PLoS One. 2023 Oct 11;18(10):e0290621. doi: 10.1371/journal.pone.0290621 (PMC10566739; doi:10.1371/journal.pone.0290621)
Supplement: S1 File — (DOCX) [file pone.0290621.s001.docx]

**Supporting Information for**

The Psychological Impacts of the COVID-19 Pandemic on Corporate Leadership

Steven Mesquiti and Sarah Seraj

Corresponding Author: Steven Mesquiti

email: [steven.mesquiti@asc.upenn.edu](mailto:steven.mesquiti@asc.upenn.edu)

**This PDF includes:**

Supplementary Text

Figure S1 to S3

Table S1 to S26

SI References

**Supplementary Text**

**This Supplementary Text File Includes:**

1. **Data Management**
2. **Content Analysis**
3. **LIWC Dimensions of Interest Text Samples**
4. **Additional LIWC Variables of Interest**
5. **Primary Statistical Tests**
6. **Post-hoc Statistical Tests**
7. **Data Management.**

After applying the exclusion criteria and obtaining the final dataset, transcripts were aligned by the month they took place (e.g., March 3, 2020 and March 29, 2020 were collapsed into March-2020). March 2019 was selected as the reference point as CEOs’ transcripts did not include mentions COVID until as early as January of 2020. Therefore, selecting a period 12 months earlier (March 2020), helped to ensure that our reference point was not influenced by discussions pertaining to the COVID-19 pandemic. LIWC scores were averaged in one-month periods by transcript. This is to ensure that we captured variation in CEOs’ language patterns across the entire period of interest. LIWC scores for analytic thinking, cognitive processes, self-focus and collective-focus were then plotted over time, which allowed us to illustrate how language changes around the pandemic (e.g. see Figure S4). We also include additional dimensions of interest in section IV.

**Figure S1**

*CEOs Discussions of COVID-19
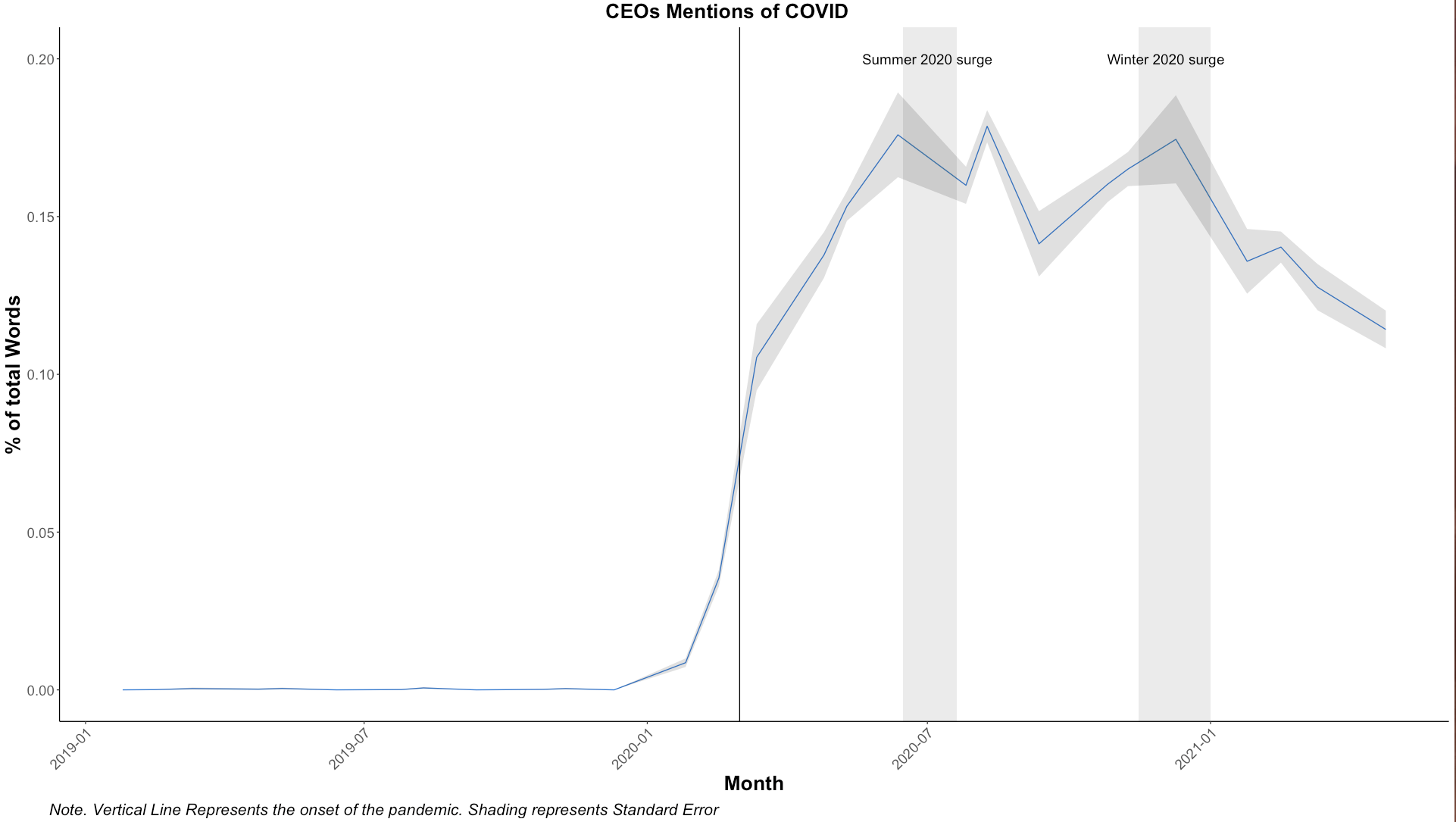
*

1. **Content Analysis**

In the main manuscript, our analyses focused on the quantitative evaluation of CEOs’ changes in function word use over time. However, it is also informative to look at content words (non-function words) and how they might have changed over time. Below is a sample of the top 25 content words within our dataset, divided into two periods: March 2019-December 2019; January 2020-March 2021 (see Table S1).

**Table S1**

*25 most frequent words in the dataset before and after the start of the pandemic.*

| March 2019-December 2019 | | January 2020-March 2021 | |
| --- | --- | --- | --- |
| Word | % of Obs | Word | % of Obs |
| year | 84.0166 | see | 88.02 |
| market | 72.4053 | time | 83.2009 |
| see | 85.5215 | year | 83.1924 |
| look | 79.7224 | look | 82.8035 |
| quarter | 74.001 | right | 82.068 |
| business | 73.4172 | business | 76.8769 |
| customers | 48.4302 | question | 76.4034 |
| growth | 64.5823 | things | 74.4758 |
| continue | 71.4971 | continue | 74.4589 |
| right | 79.0218 | quarter | 71.7535 |
| time | 78.8272 | market | 70.629 |
| years | 63.5573 | sure | 70.5952 |
| terms | 54.1905 | bit | 69.7582 |
| number | 61.6243 | said | 69.3355 |
| seeing | 57.6933 | point | 67.2049 |
| point | 63.8298 | number | 63.299 |
| mean | 54.5407 | work | 63.2398 |
| question | 69.9533 | people | 63.0115 |
| sure | 65.6201 | seeing | 62.8847 |
| great | 57.0057 | years | 61.625 |
| expect | 57.9787 | great | 61.1346 |
| thank | 55.2932 | thank | 60.5428 |
| pretty | 56.4868 | pretty | 60.1454 |
| people | 51.3622 | expect | 59.8495 |

*Note.* Word frequency is based on observation percentage (i.e. percentage of the transcripts that contain each word), not by raw frequency in the dataset.

While there was some variation in CEOs’ content word use between the two time periods, it was difficult to establish any distinct difference. To account for these differences, a factor analysis of CEO content words was conducted to extract themes and examine differences in factor scores between each time period. See below for more information on this analysis.

**Meaning Extraction Method.**

To get a feel for what CEOs were talking about before the pandemic and after its start, topic models of the dataset were constructed. Themes were extracted using a topic modeling procedure called the Meaning Extraction Method [MEM; (1)]. The MEM uses principal component analysis to mathematically calculate which groups of words are frequently uttered together, which in turn can be used to generate themes or topics that occur in a text corpus. For more information on MEM, please see Markowitz (2) and Chung and Pennebaker (1). The topic modeling tool Meaning Extraction Helper (MEH) within LIWC-22 was used to identify content words across the present dataset. A principal components analysis was then conducted on the MEH output using varimax rotation. We extracted a 3-factor solution, which provided the most cohesive themes: *Solutions, Finances, and Performance*. Table S2 contains component information for each extracted theme. Table S3 captures words that are loaded onto each corresponding theme, their eigenvalue, as well as samples of transcripts related to each theme. Table S4 shows differences in theme references across the two time periods: pre- and post-COVID. Theme scores were calculated for each transcript computing the sum of the percentage of words pertaining to each theme. That is, a theme score of 2.5% for the Finances theme indicates that 2.5% of the text contains words related to finances.

**Table S2**

*Component Summary Statistics*

| Component | 1 | 2 | 3 |
| --- | --- | --- | --- |
| Eigenvalue | 23.40 | 21.95 | 19.68 |
| %Variance accounted for | 0.03 | 0.03 | 0.03 |

**Table S3**

*Extracted Themes Loadings*

| 1 - Solutions | | 2 - Finances | | 3 - Performance | |
| --- | --- | --- | --- | --- | --- |
| data | 0.4526052282 | capital | 0.5162027182 | customers | 0.4790350793 |
| important | 0.4237666576 | cash | 0.4779017387 | growth | 0.4602954189 |
| great | 0.393067738 | sheet | 0.4511105179 | margin | 0.4499975997 |
| patience | 0.3874420579 | balance sheet | 0.4476868485 | business | 0.4437341209 |
| work | 0.3814149709 | balance | 0.4253512259 | customer | 0.4296776455 |
| excited | 0.3752510343 | debt | 0.4232141604 | products | 0.4215089922 |
| opportunity | 0.3695124469 | assets | 0.4106544259 | margins | 0.4152669893 |
| patient | 0.3654326367 | equity | 0.4078391131 | chain | 0.3982505741 |
| team | 0.3630127943 | asset | 0.3957216178 | q4 | 0.3972053749 |
| experience | 0.3605414634 | cash flow | 0.383658372 | supply chain | 0.3930870307 |
| question | 0.3571117456 | flow | 0.375288263 | supply | 0.3906153468 |
| partner | 0.3481770726 | cost | 0.3532294478 | product | 0.390432256 |
| platform | 0.3471285842 | lower | 0.3530303862 | inventory | 0.3877904448 |
| working | 0.3402338229 | basis | 0.3477031276 | demand | 0.3877103529 |
| people | 0.338138433 | low | 0.3471642849 | strong | 0.3841895864 |
| making | 0.33650049 | rates | 0.3461823376 | q2 | 0.3757418626 |
| together | 0.3319269418 | money | 0.3439784629 | q3 | 0.3714719482 |
| understand | 0.3277544255 | prices | 0.3439765139 | china | 0.3690809057 |
| access | 0.3268636612 | credit | 0.3378538354 | seeing | 0.366000302 |
| partners | 0.3247443218 | return | 0.3314366007 | quarter | 0.3646295829 |
| technology | 0.3234097662 | returns | 0.3305433177 | mix | 0.3643074094 |
| ability | 0.3218672126 | mean | 0.3299849565 | performance | 0.3620940002 |
| time | 0.3213609649 | pretty | 0.3266578406 | q1 | 0.3563832161 |
| things | 0.3194160669 | pay | 0.3254664249 | sales | 0.3526264663 |
| provide | 0.3186226842 | higher | 0.3252574917 | half | 0.3484303459 |
| bring | 0.31818224 | price | 0.3202265543 | drive | 0.3469036553 |
| early | 0.3167520395 | rate | 0.3189488133 | improvement | 0.3417039635 |
| launch | 0.3149772435 | risk | 0.3145313417 | driving | 0.3413355983 |
| care | 0.3141620088 | long | 0.3132674385 | continue | 0.3357336973 |
| fact | 0.3116842522 | bit | 0.3130408966 | market | 0.3342970398 |
| talk | 0.3108437437 | amount | 0.3112048979 | north | 0.3327000518 |
| sure | 0.3078816085 | costs | 0.3109924313 | grow | 0.3314423411 |
| set | 0.3000685221 | stock | 0.3002941405 | growing | 0.3274678846 |
|  |  |  |  | volume | 0.3264327047 |
|  |  |  |  | impact | 0.3243121547 |
|  |  |  |  | market | 0.3239249273 |
|  |  |  |  | America | 0.3237797928 |
|  |  |  |  | revenue | 0.3218175007 |
|  |  |  |  | year | 0.3213239374 |

*Note.* Values displayed are eigenvalues. We only included words that loaded onto each factor with an eigenvalue > 0.30

**Examples of transcripts highly related to each theme**

**Solutions:**

Ralph, please? Next question, please? Very good question. Ralph? Thank you. Operator, are there any other questions at this time? Do you want instruct once again how people are going to ask questions? Ralph? John asked a tricky question… But it’s good. Only the non-Israeli patients pay. The whole – reason why, the Israeli government wanted us to treat Israeli patients which can’t afford anything. So they are paying zero. And the Israeli Medical Tourism opportunity allows us to treat a few patients in Israel for free. That was in return of not having an arm of the trial in Israel. Both the trial would cost. A few of them, exactly, five patients support the other eight. 100% and I think these patients are very good-hearted people, not only as they want to get possibility of treatments, they really are very happy that other people are also getting this opportunity and so, the right time to thank them too. Thank you. Any other questions? Thank you very much. Operator, you can go and proceed to the closing comment.

**Finances:**

Sure. Yes. At sub 30 prices the hedging does allow- provide enough cash flow to cover our overhead G&A interest expense so forth. Obviously, as discussed at $30 prices unless the differential is out of line would be bringing production back on. But and then at lower WTI prices, the hedges actually would generate even more income. So as Kelly said short answer is yes, the hedges would provide enough cash flow to cover our overhead.

**Performance:**

Sure. Hi, Debra. Yes. Last year, 2019 was a great year for us at equipment sales. 2020, I think and expect to be also a good year, probably slightly down because one of our large customers in '19 won't be repurchasing. Equipment sales are somewhat cyclical. But I expect a good year in 2020 as well. Gallon, then service revenue.

**Table S4**

*Comparing variance of themes across the time periods*

|  | Mean % of words | |  |  |  |
| --- | --- | --- | --- | --- | --- |
| Theme | Pre-COVID | Post-COVID | T-value | df | Cohen’s D |
| Solutions | 1.9% | 2% | -10.341*** | 19,514 | -0.148 |
| Finances | 1.4% | 1.3% | 5.812*** | 19,467 | 0.083 |
| Performance | 3.1% | 2.7% | 15.966*** | 19,521 | 0.228 |

*Note. *** p* < 0.001

Next, we conducted paired t-tests to observe how our constructed themes might have changed as a function of the pandemic. The results from our analyses (see Table S4) suggest that there is a thematic difference in the earnings calls transcripts across the two time periods. It appears that CEOs before the pandemic were more focused on financial topics and the performance of their companies. However, we see an increase in their interest in crafting solutions in calls that were conducted after the start of the pandemic. Our results suggest CEOs' focus in these calls shifted from interpreting financial reports to actively discussing strategy on these calls, consistent with our main findings.

1. **LIWC Dimensions of Interest Text Samples**

**Table S5**

*LIWC Dimensions of Interest Samples*

| LIWC Dimension | Score | Example Text |
| --- | --- | --- |
| Analytic Thinking | High | **I** mean **the** total number **of** shares **authorized** **by** **the** Board **at** March 31 **was** 7.2 million shares **that** **were** left, a **considerable** amount left. |
|  | Low | **No**, **you** are absolutely right. **The** studies **have** been done and shown that **there** really **isn't** congestion. So, **we** have **no** concerns **in** that regard. |
| Cognitive Processing | High | Let me have, Ulrich, **maybe** you **can** specifically **respond** to those parameters that Jason is asking. Great **question**. Again Ulrich, **would** you take that please? |
|  | Low | Thank you, very much for your continued interest in our company and we look forward to speaking with you again in our next earnings call. |
| We-words | High | **We** want to thank **our** investors, **our** employees and **our** partners and **our** customers for their interest in **our** company and **we** look forward to reporting **our** second quarter results at the end of July, 2015. Thank you. |
|  | Low | Yes, and Josh, this is JP. I would just add price exactly right. There was a 10b5-1 plan that was written and implemented back last year. I have not had a window open to me to change it. That is the fundamental. And I've told investors who called me and asked me that I've spoken to them directly about it. It's not something that I am legally allowed to change until a window opens. I do intend to make changes to it when the window opens, but I can't do it until then. |
| I-words | High | And Marni, this is Michael. Yes, you did hear correctly. **I** will do that in addition to **my** other responsibilities, and **I** hope **I** can be up to this task. |
|  | Low | Yeah, we should see some benefit from that, yes. We haven't gone that granular as far as the timing of that shipment. Thank you, Jeff. |
| COVID- mentions | High | Q4 was a solid quarter for us and we began 2020 with great momentum. But given the evolving **coronavirus** situation we're not going to give guidance in 2020 at this point. What is true is there **coronavirus** is not going to stop fraud, that's for sure. |
|  | Low | No, you are absolutely right. The studies have been done and shown that there really isn't congestion. So, we have no concerns in that regard. |

*Note*. Words in red result in higher scores for the respective LIWC Dimension. Analytic thinking is the only LIWC dimension presented that is a bidirectional dimension. The words highlighted in blue would cause analytic thinking scores to be lower. For the other three categories, higher scores indicate that a greater % of words pertaining to that category was present in the sample.

1. **Additional LIWC Variables of Interest**

Although the main paper focused on four language dimensions within LIWC-22, there were several other dimensions that may shed light on additional cognitive processes. Patterns of interest across other linguistic categories are provided below for the following areas: Emotion, Focus, and LIWC Summary Variables.

**Affect**

Emotions are linked to the way individuals view their world and use of emotion words captures their unique perception of the world (3, 4). In particular, language research on social upheavals has shown that negative emotion word usage is often highest when individuals are in the midst of experiencing a distressing event (5, 6).

**Figure S1**

*Graphical Representation of Emotion Language Change*


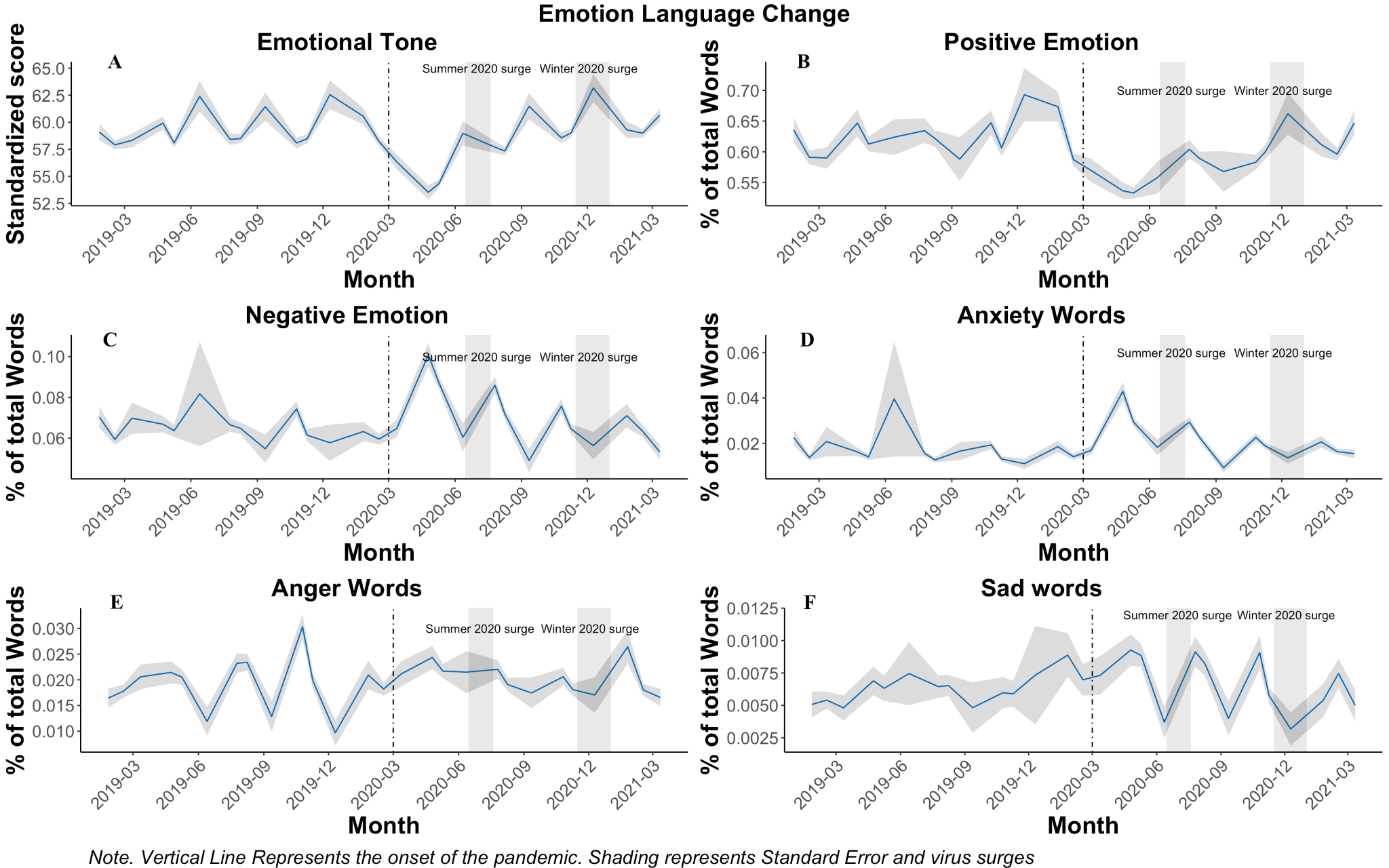


Within the context of business leaders, CEOs are often under a great deal of external and internal pressure to successfully lead their companies. As a result, they may experience strong shifts in their emotions. To gain a better understanding of how the onset of pandemic affected the emotional valence of CEOs, we plotted the LIWC variable Emotional Tone throughout our period of interest, along with other emotional categories (Figure S1). Within LIWC-22, the dimension Emotional Tone is the difference between positive and negative emotion words within a text (7). Higher levels of Emotional Tone indicate a message with more positive valence. At the start of the pandemic, CEOs’ tone was at its lowest, suggesting that the most extreme emotions occurred once lockdown began. We also observed the sharpest increase in anxiety words usage at the beginning of the pandemic. This increase in negative emotional tone and anxiety words at the time of the pandemic’s start, suggests that pandemic placed CEOs’ under a great deal of stress. While it improved as time progressed it did not return to pre-pandemic levels. Of note is that the strong shift in negative emotion words share similar cyclical trends with self-focus language (I-words), both of which are linked to things like stress and depression (8).

**Focus**

In addition to emotion words, we explored the temporal focus of CEOs by examining their use of past, present, and future tense words. Past research has shown that verb tense differences may act as a proxy to indicate psychological distance and attention (9). Studying *where* one’s focus is may give us a deeper understanding of the ways people process their environment.

**Figure S2**

*Graphical Representation of Temporal Focus Language Change*


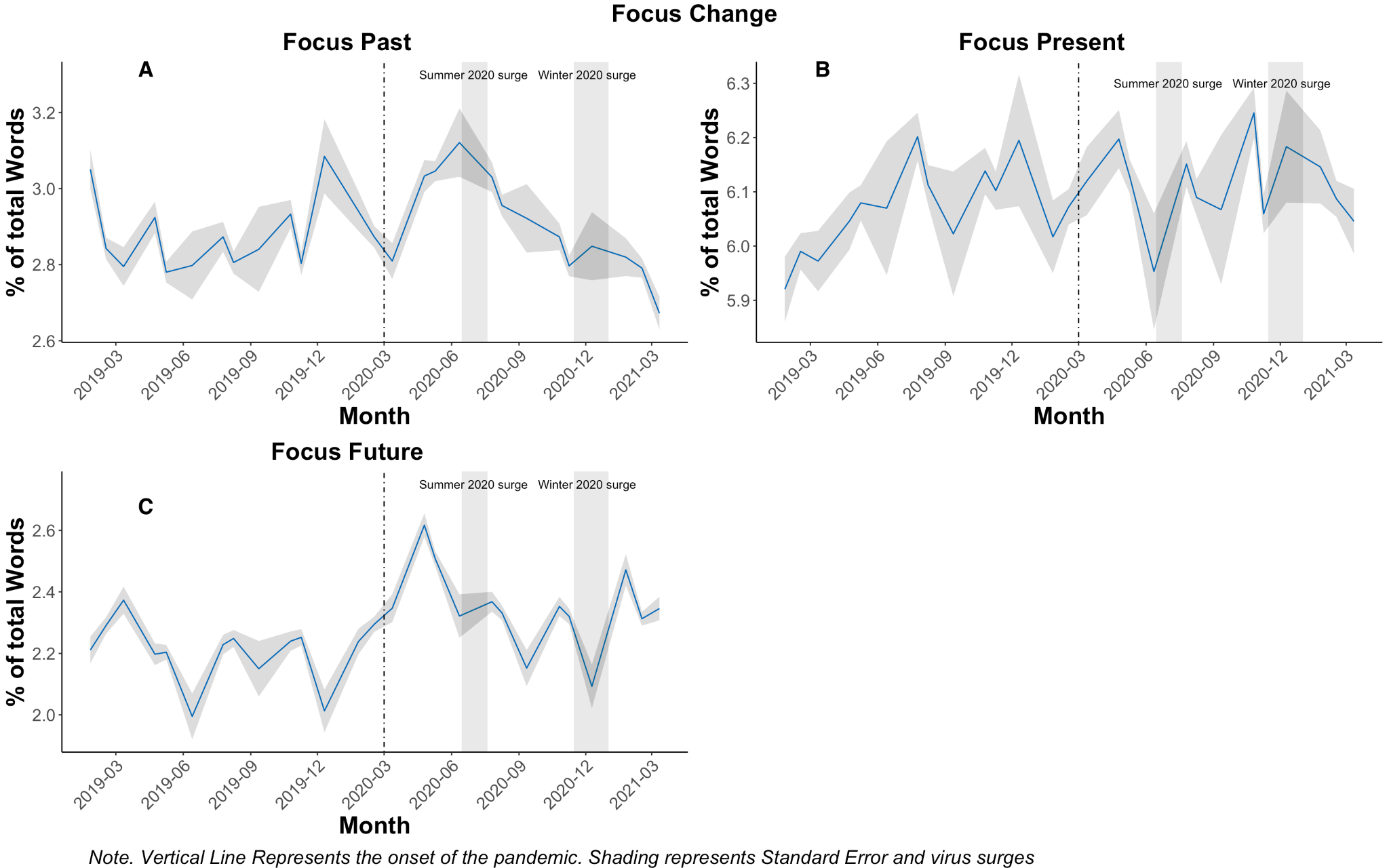


To better understand how CEOs’ temporal focus may have changed after the onset of pandemic, we plotted the past, present, and future tense dimensions through our period of interest (Figure S2). At the start of the pandemic, we observed sharp increases in CEOs’ past and future-focused language; however, we did not observe similar shifts in present-focused language. The increase in past tense words after the pandemic’s start, suggests that CEOs may have repeatedly cited past events (e.g., the Great Recession). Increases in future tense verbs may corroborate this. Specifically, CEOs may have been forecasting how their companies would respond to the economy’s crash, a finding that adds to our research questions.

**LIWC Summary Variables**

LIWC summary variables, which are meant to give a big picture overview of people’s language, were also plotted to see how CEOs’ attention towards others changed. The LIWC summary variable, Clout, measures relational attention through people’s pronoun use (9,10), as clout and first-person plural pronouns (e.g., our, us) are positively related. The decrease in clout levels immediately after the pandemic could be the result of CEOs’ decreases in collective-focus language and may not add any further insight in our research questions.

We also examined other summary variables within LIWC because of their link to cognitive processes. Research has found that individuals who use more complex language (e.g., use of 6+ letter words and words per sentence), in their writing tend to be in better cognitive health (11). All or none language pertains to absolutist language or “black-and-white” speak, indicative of less nuanced thinking. Decreases in business executives’ complex language after the pandemic suggests an inability to effectively organize their thoughts. Increases in all or none language after the pandemic suggests that CEOs may have been attempting to take more extreme positions regarding topics surrounding their companies.

**Figure S3**

*Changes in LIWC Summary Variables*

*
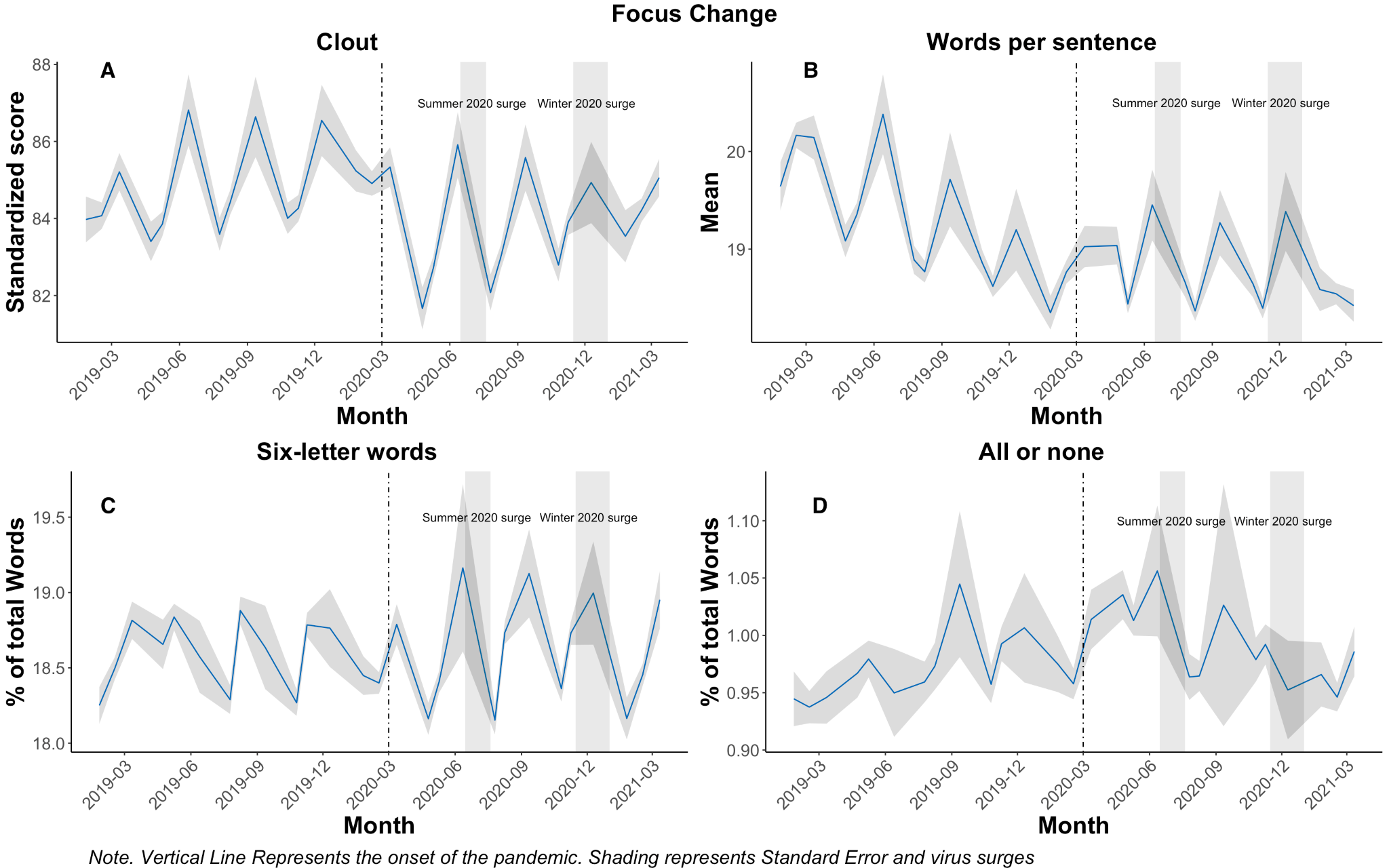
*

**V. Primary Statistical Tests**

**Table S6**

*T-tests between each month period and time zero (March 2019) for Analytic thinking words.*

| Time period | | Analytic Thinking | | | |
| --- | --- | --- | --- | --- | --- |
| T1 | T2 | t-test | p-value | Cohen’s D | Mean diff |
| Time Zero | April 2019 | t(1161.5) = 1.5025 | 0.1332 | 0.08712894 | 1.3114081 |
| Time Zero | May 2019 | t(1036.8) = 0.68601 | 0.4929 | 0.03299797 | 0.4935284 |
| Time Zero | June 2019 | t(245.14) = 0.25079 | 0.8022 | 0.02061074 | 0.3039970 |
| Time Zero | July 2019 | t(1120.1) = 2.6728 | 0.007631 | 0.1587215 | 2.3251490 |
| Time Zero | August 2019 | t(1004.8) = 0.47855 | 0.6324 | 0.02352348 | 0.3411544 |
| Time Zero | September 2019 | t(280.43) = 1.0343 | 0.3019 | 0.08670448 | 1.3027809 |
| Time Zero | October 2019 | t(1049.9) = 2.6675 | 0.00776 | 0.1620807 | 2.3954214 |
| Time Zero | November 2019 | t(993.35) = 1.4046 | 0.1605 | 0.06871468 | 0.9976299 |
| Time Zero | December 2019 | t(328.09) = 1.0147 | 0.311 | 0.0805849 | 1.1986758 |
| Time Zero | January 2020 | t(286.24) = 1.5505 | 0.1221 | 0.1282654 | 1.9188652 |
| Time Zero | February 2020 | t(1061.6) = 1.9738 | 0.04867 | 0.1023933 | 1.4369448 |
| Time Zero | March 2020 | t(1272.1) = 1.3054 | 0.192 | 0.06944158 | 1.0438407 |
| Time Zero | April 2020 | t(623.94) = 5.777 | 1.201e-08 | 0.3954264 | 5.7784625 |
| Time Zero | May 2020 | t(929.48) = 5.1516 | 3.153e-07 | 0.2534133 | 3.5880071 |
| Time Zero | June 2020 | t(370.16) = 1.4219 | 0.1559 | 0.1138341 | 1.7436794 |
| Time Zero | July 2020 | t(316.92) = 3.9258 | 0.0001061 | 0.3057368 | 4.4919977 |
| Time Zero | August 2020 | t(918.09) = 3.2572 | 0.001166 | 0.1588173 | 2.2602447 |
| Time Zero | September 2020 | t(302.23) = 0.11712 | 0.9068 | 0.01015581 | 0.1589776 |
| Time Zero | October 2020 | t(164.425) = 0.84629 | 0.3986 | 0.0861013 | 1.3178462 |
| Time Zero | November 2020 | t(920.44) = 3.7364 | 0.0001981 | 0.180298 | 2.5943085 |
| Time Zero | December 2020 | t(331.79) = 0.63931 | 0.5231 | 0.05298193 | 0.8151869 |
| Time Zero | January 2021 | t(63.201) = 2.6168 | 0.01109 | 0.323724 | 4.8802673 |
| Time Zero | February 2021 | t(1112) = 3.7687 | 0.0001727 | 0.201862 | 2.8046380 |
| Time Zero | March 2021 | t(1125.2) = 2.4326 | 0.01515 | 0.1262979 | 1.8105501 |

**Table S7**

*Welch’s t-tests between each month after the pandemic and the following month (March 2020-March 2021) for Analytic Thinking in the CEO dataset*

| Time period | | Analytic Thinking | | | |
| --- | --- | --- | --- | --- | --- |
| T | T+1 | t-test | p-value | Cohen’s D | Mean diff |
| Mar 2020 | Apr 2020 | t(525.79) = 5.0849 | 5.124e-07 | 0.3274589 | 4.734622 |
| Apr 2020 | May 2020 | t(373.06) = -2.5948 | 0.009839 | -0.1597933 | -2.190455 |
| May 2020 | Jun 2020 | t(252.04) = -1.6726 | 0.09565 | -0.1320224 | -1.844328 |
| Jun 2020 | Jul 2020 | t(377.62) = 1.9242 | 0.05508 | 0.1935631 | 2.748318 |
| Jul 2020 | Aug 2020 | t(200.57) = -2.2122 | 0.02808 | -0.1616992 | -2.231753 |
| Aug 2020 | Sept 2020 | t(218.93) = -1.6872 | 0.09298 | -0.1481301 | -2.101267 |
| Sept 2020 | Oct 2020 | t(262.61) = 0.61994 | 0.5358 | 0.07097007 | 1.158869 |
| Oct 2020 | Nov 2020 | t(128.22) =0.87379 | 0.3839 | 0.08987477 | 1.276462 |
| Nov 2020 | Dec 2020 | t(230.76) = -1.5398 | 0.125 | -0.1246402 | -1.779122 |
| Dec 2020 | Jan 2021 | t(94.317) = 1.9533 | 0.05374 | 0.2681803 | 4.06508 |
| Jan 2021 | Feb 2021 | t(55.552) = -1.1498 | 0.2552 | -0.1598304 | -2.075629 |
| Feb 2021 | Mar 2021 | t(2141.4) = -1.7179 | 0.08596 | -0.07394617 | -0.994088 |

**Table S8**

*T-tests between each month period and time zero (March 2019) for Cognitive processing words.*

| Time period | | Cognitive processing words | | | |
| --- | --- | --- | --- | --- | --- |
| T1 | T2 | t-test | p-value | Cohen’s D | Mean diff |
| Time Zero | April 2019 | t(1156.5) = -0.50972 | 0.6103 | -0.02989588 | -0.06714981 |
| Time Zero | May 2019 | t(1036) = -0.71786 | 0.473 | -0.03454592 | -0.07608954 |
| Time Zero | June 2019 | t(218.72) = -0.23913 | 0.8112 | -0.02131939 | -0.04722150 |
| Time Zero | July 2019 | t(1119.7) = -1.8417 | 0.06579 | -0.1093919 | -0.23650769 |
| Time Zero | August 2019 | t(1051.9) = -0.37635 | 0.7067 | -0.01803689 | -0.04007954 |
| Time Zero | September 2019 | t(282.79) = 0.24423 | 0.8072 | 0.02036127 | 0.04500480 |
| Time Zero | October 2019 | t(1029.2) = -1.7142 | 0.0868 | -0.1048291 | -0.23360258 |
| Time Zero | November 2019 | t(1076.6) = -0.95381 | 0.3404 | -0.04459361 | -0.10223869 |
| Time Zero | December 2019 | t(320.31) = 1.0446 | 0.297 | 0.08411213 | 0.18530265 |
| Time Zero | January 2020 | t(255.26) = -0.81688 | 0.4148 | -0.07319062 | -0.16607954 |
| Time Zero | February 2020 | t(1147.6) = -0.72454 | 0.4689 | -0.03642406 | -0.07979527 |
| Time Zero | March 2020 | t(1307.9) = -2.028 | 0.04276 | -0.1070381 | -0.24317116 |
| Time Zero | April 2020 | t(609.25) = -5.7012 | 1.855e-08 | -0.3938811 | -0.85389986 |
| Time Zero | May 2020 | t(924.04) = -6.5911 | 7.329e-11 | -0.3255788 | -0.67542245 |
| Time Zero | June 2020 | t(395.99) = -0.38556 | 0.7 | -0.02978281 | -0.06597201 |
| Time Zero | July 2020 | t(298.22) = -4.0812 | 5.758e-05 | -0.3291694 | -0.71999520 |
| Time Zero | August 2020 | t(949) = -5.465 | 5.916e-08 | -0.260103 | -0.56401082 |
| Time Zero | September 2020 | t(310.67) = 0.92648 | 0.3549 | 0.07887731 | 0.18019670 |
| Time Zero | October 2020 | t(184.74) = -0.57971 | 0.5628 | -0.05270504 | -0.11602032 |
| Time Zero | November 2020 | t(936.81) = -3.7994 | 0.000154 | -0.1809343 | -0.39065633 |
| Time Zero | December 2020 | t(341.61) = 0.7639 | 0.4455 | 0.06221595 | 0.13984154 |
| Time Zero | January 2021 | t(61.973) = -1.382 | 0.1719 | -0.1777619 | -0.39595552 |
| Time Zero | February 2021 | t(1140) = -1.0691 | 0.2853 | -0.05682646 | -0.11836780 |
| Time Zero | March 2021 | t(1172.3) = -1.8593 | 0.06323 | -0.09512648 | -0.20702776 |

**Table S9**

*Welch’s t-tests between each month after the pandemic and the following month (March 2020-March 2021) for Cognitive processing words in the CEO dataset*

| Time period | | Cognitive Processing | | | |
| --- | --- | --- | --- | --- | --- |
| T | T+1 | t-test | p-value | Cohen’s D | Mean diff |
| Mar 2020 | Apr 2020 | t(534.57) = -4.3161 | 1.893e-05 | -0.2755415 | -0.61072870 |
| Apr 2020 | May 2020 | t(366.54) = 1.4046 | 0.161 | 0.0887056 | 0.17847741 |
| May 2020 | Jun 2020 | t(257.87) = 4.0193 | 7.665e-05 | 0.3007241 | 0.60945044 |
| Jun 2020 | Jul 2020 | t(367.3) = -3.1317 | 0.001877 | -0.3204553 | -0.65402320 |
| Jul 2020 | Aug 2020 | t(199.24) = 0.98679 | 0.3249 | 0.07325564 | 0.15598439 |
| Aug 2020 | Sept 2020 | t(223.61) = 4.1804 | 4.178e-05 | 0.3435609 | 0.74420751 |
| Sept 2020 | Oct 2020 | t(285.88) = -1.1984 | 0.2318 | -0.1329353 | -0.29621702 |
| Oct 2020 | Nov 2020 | t(133.62) = -1.493 | 0.1378 | -0.1294167 | -0.27463600 |
| Nov 2020 | Dec 2020 | t(234.85) = 3.2109 | 0.001508 | 0.2476709 | 0.53049787 |
| Dec 2020 | Jan 2021 | t(87.346) = -1.7045 | 0.09183 | -0.2453381 | -0.53579706 |
| Jan 2021 | Feb 2021 | t(55.376) = 0.99678 | 0.3232 | 0.1405453 | 0.27758771 |
| Feb 2021 | Mar 2021 | t(2145.1) = -0.99943 | 0.3177 | -0.04297582 | -0.08865995 |

**Table S10**

*T-tests between each month period and time zero (March 2019) for Collective-Focus words.*

| Time period | | Collective-Focus | | | |
| --- | --- | --- | --- | --- | --- |
| T1 | T2 | t-test | p-value | Cohen’s D | Mean diff |
| Time Zero | April 2019 | t(1161.9) = 0.57178 | 0.5676 | 0.03344116 | 0.05307349 |
| Time Zero | May 2019 | t(1008.4) = 1.5919 | 0.1117 | 0.07777732 | 0.12266397 |
| Time Zero | June 2019 | t( 214.75) = -1.0685 | 0.2865 | -0.09667541 | -0.15750234 |
| Time Zero | July 2019 | t(1116.2) = 0.61537 | 0.5384 | 0.03621198 | 0.05448333 |
| Time Zero | August 2019 | t( 979.1) = 0.93964 | 0.3476 | 0.04688513 | 0.07179231 |
| Time Zero | September 2019 | T(280.32) = -1.1796 | 0.2392 | -0.09890573 | -0.16049082 |
| Time Zero | October 2019 | t(1067.9)= -0.20364 | 0.8387 | -0.01227638 | -0.01908528 |
| Time Zero | November 2019 | t(972.54) = 0.64972 | 0.516 | 0.03219267 | 0.04953027 |
| Time Zero | December 2019 | t( 353.8) = -0.63075 | 0.5286 | -0.04825792 | -0.07655307 |
| Time Zero | January 2020 | t(309.04) = -0.96769 | 0.334 | -0.07643709 | -0.12175588 |
| Time Zero | February 2020 | t(1073.8) = -0.92675 | 0.3543 | -0.04785233 | -0.07312741 |
| Time Zero | March 2020 | t(1197.2) = -0.40017 | 0.6891 | -0.02162594 | -0.03345200 |
| Time Zero | April 2020 | t( 676.59) = 3.3604 | 0.000822 | 0.2228626 | 0.34434120 |
| Time Zero | May 2020 | t( 890.34) = 5.6601 | 2.04e-08 | 0.287374 | 0.42067923 |
| Time Zero | June 2020 | t(395.82) = 0.42318 | 0.6724 | 0.03269633 | 0.05307466 |
| Time Zero | July 2020 | t(317.82) = 3.3898 | 0.0007876 | 0.2635803 | 0.41800316 |
| Time Zero | August 2020 | t( 889.2) = 5.1356 | 3.457e-07 | 0.2566654 | 0.38147968 |
| Time Zero | September 2020 | t(361.98) = -0.71644 | 0.4742 | -0.05574824 | -0.08957540 |
| Time Zero | October 2020 | t(191.38) = 2.3094 | 0.02199 | 0.2039772 | 0.32728027 |
| Time Zero | November 2020 | t(873.54) = 4.1802 | 3.205e-05 | 0.2102911 | 0.30899565 |
| Time Zero | December 2020 | t(390.06) = 0.86669 | 0.3866 | 0.06570682 | 0.10483027 |
| Time Zero | January 2021 | t(64.772) = 0.22875 | 0.8198 | 0.02706886 | 0.04394693 |
| Time Zero | February 2021 | t(1081.1) = 2.5427 | 0.01114 | 0.1373736 | 0.20223575 |
| Time Zero | March 2021 | t(1081) = 2.2873 | 0.02237 | 0.1204946 | 0.18128027 |

**Table S11**

*Welch’s t-tests between each month after the pandemic and the following month (March 2020-March 2021) for Collective-focus words in the CEO dataset*

| Time period | | Collective-focus | | | |
| --- | --- | --- | --- | --- | --- |
| T | T+1 | t-test | p-value | Cohen’s D | Mean diff |
| Mar 2020 | Apr 2020 | t(527.08) = 4.1038 | 4.709e-05 | 0.2639367 | 0.37779320 |
| Apr 2020 | May 2020 | t(378.82) = 0.9117 | 0.3625 | 0.05499341 | 0.07633803 |
| May 2020 | Jun 2020 | t(253.14) = -3.3226 | 0.001023 | -0.2594704 | -0.36760457 |
| Jun 2020 | Jul 2020 | t(373.96) = 2.4647 | 0.01416 | 0.2501259 | 0.36492850 |
| Jul 2020 | Aug 2020 | t(197.52) = -0.33751 | 0.7361 | -0.02558748 | -0.03652348 |
| Aug 2020 | Sept 2020 | t(229.5) = -4.2759 | 2.794e-05 | -0.3276203 | -0.47105508 |
| Sept 2020 | Oct 2020 | t(262.6) = 2.551 | 0.01131 | 0.2920369 | 0.41685567 |
| Oct 2020 | Nov 2020 | t(131.79) = -0.1422 | 0.8871 | -0.01296356 | -0.01828462 |
| Nov 2020 | Dec 2020 | t(238.21) = -0.1422 | 0.05362 | -0.1443587 | -0.20416538 |
| Dec 2020 | Jan 2021 | t(84.062) = -0.29524 | 0.7685 | -0.04359994 | -0.06088333 |
| Jan 2021 | Feb 2021 | t(55.764) = 0.85566 | 0.3958 | 0.1169953 | 0.15828882 |
| Feb 2021 | Mar 2021 | t(2137.8) = -0.34954 | 0.7267 | -0.01505727 | -0.02095549 |

**Table S12**

*T-tests between each month period and time zero (March 2019) for Self-focus words.*

| Time period | | Self-focus | | | |
| --- | --- | --- | --- | --- | --- |
| T1 | T2 | t-test | p-value | Cohen’s D | Mean diff |
| Time Zero | April 2019 | t(1143.8) = -3.345 | 0.0008495 | -0.1965974 | -0.17476699 |
| Time Zero | May 2019 | t(1155.2) = -1.1963 | 0.2318 | -0.05439808 | -0.05043041 |
| Time Zero | June 2019 | t(213.55) = -0.19114 | 0.8486 | -0.017372 | -0.01487742 |
| Time Zero | July 2019 | t(1114.3) = -4.1439 | 3.672e-05 | -0.2467407 | -0.20822325 |
| Time Zero | August 2019 | t(1056.6) = -0.64768 | 0.5173 | -0.03096763 | -0.02656967 |
| Time Zero | September 2019 | t(278.03) = -1.6111 | 0.1083 | -0.1358241 | -0.11592509 |
| Time Zero | October 2019 | t(1035.2) = -3.3533 | 0.0008274 | -0.2047181 | -0.17440793 |
| Time Zero | November 2019 | t(1067) = -2.0582 | 0.03981 | -0.09669761 | -0.08464259 |
| Time Zero | December 2019 | t(265.19) = -1.4168 | 0.1577 | -0.1296303 | -0.11621559 |
| Time Zero | January 2020 | t(284.3) = -2.7747 | 0.005891 | -0.2305339 | -0.19580461 |
| Time Zero | February 2020 | t(1154.3) = -1.9849 | 0.04739 | -0.09955451 | -0.08426835 |
| Time Zero | March 2020 | t(1263.5) = -0.332 | 0.7399 | -0.01769369 | -0.01499184 |
| Time Zero | April 2020 | t(571.49) = -5.028 | 6.644e-07 | -0.3562055 | -0.30279623 |
| Time Zero | May 2020 | t(958.88) = -3.7093 | 0.0002198 | -0.1785725 | -0.14774293 |
| Time Zero | June 2020 | t(390.58) = 0.22143 | 0.8249 | 0.01722659 | 0.01473249 |
| Time Zero | July 2020 | t(253.44) = -3.9255 | 0.0001116 | -0.3536629 | -0.30941907 |
| Time Zero | August 2020 | t(1005.4) = -4.4733 | 8.58e-06 | -0.2047237 | -0.18049992 |
| Time Zero | September 2020 | t(350.62) = 0.4135 | 0.6795 | 0.03273799 | 0.02781419 |
| Time Zero | October 2020 | t(180.6) = -2.646 | 0.008864 | -0.2453415 | -0.20855834 |
| Time Zero | November 2020 | t(986.11) = -4.3779 | 1.326e-05 | -0.2010721 | -0.17565668 |
| Time Zero | December 2020 | t(371.13) = -1.3222 | 0.1869 | -0.1028381 | -0.08706003 |
| Time Zero | January 2021 | t(63.336) = 1.3508 | 0.1816 | 0.1664219 | 0.14220271 |
| Time Zero | February 2021 | t(1250.8) = -5.6223 | 2.322e-08 | -0.2903836 | -0.24899238 |
| Time Zero | March 2021 | t(1254.8) = -1.8931 | 0.05858 | -0.09454125 | -0.08328284 |

**Table S13**

*Welch’s t-tests between each month after the pandemic and the following month (March 2020-March 2021) for Self-focus words in the CEO dataset*

| Time period | | Self-focus | | | |
| --- | --- | --- | --- | --- | --- |
| T | T+1 | t-test | p-value | Cohen’s D | Mean diff |
| Mar 2020 | Apr 2020 | t(477.85) = -5.1026 | 4.842e-07 | -0.3467518 | -0.28780439 |
| Apr 2020 | May 2020 | t(362.97) = 2.9683 | 0.003194 | 0.1902125 | 0.15505330 |
| May 2020 | Jun 2020 | t(261.2) = 2.7352 | 0.006661 | 0.1990807 | 0.16247542 |
| Jun 2020 | Jul 2020 | t(336.98) = -3.5895 | 0.0003805 | -0.3757604 | -0.32415156 |
| Jul 2020 | Aug 2020 | t(191.52) = 1.7614 | 0.07976 | 0.1451672 | 0.12891915 |
| Aug 2020 | Sept 2020 | t(240.73) = 3.4394 | 0.000687 | 0.2369631 | 0.20831411 |
| Sept 2020 | Oct 2020 | t(255.11) = -2.6019 | 0.009813 | -0.3007221 | -0.23637253 |
| Oct 2020 | Nov 2020 | t(134.91) = 0.45032 | 0.6532 | 0.0377993 | 0.03290166 |
| Nov 2020 | Dec 2020 | t(248.77) = 1.5059 | 0.1334 | 0.1020099 | 0.08859665 |
| Dec 2020 | Jan 2021 | t(84.284) = 2.0159 | 0.047 | 0.2971566 | 0.22926275 |
| Jan 2021 | Feb 2021 | t(57.559) = -3.8068 | 0.0003437 | -0.4621942 | -0.39119509 |
| Feb 2021 | Mar 2021 | t(2135.8) = 4.4095 | 1.088e-05 | 0.1900173 | 0.16570954 |

Table S14

*Welch’s t-tests between each month after the pandemic and the following month (March 2020-March 2021) for Analytic Thinking in the Reddit dataset*

| Time period | |  | | Analytic Thinking | | | | | | |
| --- | --- | --- | --- | --- | --- | --- | --- | --- | --- | --- |
| T1 | | T2 | | t-test | | p-value | | Cohen’s d | | Mean diff |
| Mar 2020 | Apr 2020 | | t(718082.08) = 8.29 | | 1.130383e-16 | | 0.0195406 | | 0.5773111 | |
| Apr 2020 | May 2020 | | t(723659.736) = -11.166 | | 5.992943e-29 | | -0.0262343 | | -0.7725293 | |
| May 2020 | Jun 2020 | | t(764139.358) = -6.811 | | 9.714487e-12 | | -0.0147527 | | -0.4363444 | |
| Jun 2020 | Jul 2020 | | t(739955.958) = -5.062 | | 4.155963e-07 | | -0.0110586 | | -0.3271750 | |
| Jul 2020 | Aug 2020 | | t(733398.594) = -8.979 | | 2.726329e-19 | | -0.0207349 | | -0.6113902 | |
| Aug 2020 | Sept 2020 | | t(714206.373) = 0.791 | | 4.290524e-01 | | 0.0018421 | | 0.0543964 | |
| Sept 2020 | Oct 2020 | | t(574976.112) = -2.535 | | 1.124332e-02 | | -0.0065697 | | -0.1942589 | |
| Oct 2020 | Nov 2020 | | t(553449.881) = 5.313 | | 1.079432e-07 | | 0.0142587 | | 0.4219028 | |
| Nov 2020 | Dec 2020 | | t(543229.425) = -1.814 | | 6.961340e-02 | | -0.0049063 | | -0.1452500 | |
| Dec 2020 | Jan 2021 | | t(526558.987) = 2.678 | | 7.413596e-03 | | 0.0073793 | | 0.2187492 | |
| Jan 2021 | Feb 2021 | | t(406785.341) = -7.035 | | 1.998547e-12 | | -0.0211711 | | -0.6276734 | |
| Feb 2021 | Mar 2021 | | t(401150.438) = 2.145 | | 3.199039e-02 | | 0.0066900 | | 0.1978286 | |

Table S15

*Welch’s t-tests between each month after the pandemic and the following month (March 2020-March 2021) for Cognitive Processing in the Reddit dataset*

| Time period | | Cognitive Processing | | | | |
| --- | --- | --- | --- | --- | --- | --- |
| T1 | T2 | | t-test | p-value | Cohen’s d | Mean diff |
| Mar 2020 | Apr 2020 | | t(717480.372) = -2.806 | 5.012362e-03 | -0.0066155 | -0.0405649 |
| Apr 2020 | May 2020 | | t(723590.442) = -7.439 | 1.015731e-13 | -0.0174776 | -0.1065350 |
| May 2020 | Jun 2020 | | t(772303.076) = -15.24 | 1.948292e-52 | -0.0329073 | -0.2035422 |
| Jun 2020 | Jul 2020 | | t(746859.81) = 7.027 | 2.106947e-12 | 0.0153100 | 0.0948325 |
| Jul 2020 | Aug 2020 | | t(733615.996) = 7.685 | 1.534463e-14 | 0.0177439 | 0.1087069 |
| Aug 2020 | Sept 2020 | | t(714215.747) = 6.826 | 8.728230e-12 | 0.0159007 | 0.0975957 |
| Sept 2020 | Oct 2020 | | t(574551.706) = -3.116 | 1.834050e-03 | -0.0080765 | -0.0496766 |
| Oct 2020 | Nov 2020 | | t(553413.335) = 2.184 | 2.899172e-02 | 0.0058604 | 0.0360969 |
| Nov 2020 | Dec 2020 | | t(543593.836) = 4.957 | 7.154611e-07 | 0.0134022 | 0.0824347 |
| Dec 2020 | Jan 2021 | | t(526648.095) = -2.858 | 4.266607e-03 | -0.0078751 | -0.0485719 |
| Jan 2021 | Feb 2021 | | t(409613.179) = 13.104 | 3.197581e-39 | 0.0393576 | 0.2424028 |
| Feb 202 | Mar 2021 | | t(401585.332) = -6.871 | 6.366251e-12 | -0.0214298 | -0.1309687 |

**Table S16**

*Welch’s t-tests between each month after the pandemic and the following month (March 2020-March 2021) for Self-focused language in the Reddit dataset*

| Time period | | Self-focused | | | |
| --- | --- | --- | --- | --- | --- |
| T1 | T2 | t-test | p-value | Cohen’s d | Mean diff |
| Mar 2020 | Apr 2020 | t(719466.558) = -6.242 | 4.321693e-10 | -0.0147050 | -0.0552501 |
| Apr 2020 | May 2020 | t(724928.553) = 13.029 | 8.442554e-39 | 0.0305890 | 0.1137677 |
| May 2020 | Jun 2020 | t(721876.294) = 48.949 | 0.000000e+00 | 0.1077292 | 0.3775029 |
| Jun 2020 | Jul 2020 | t(721351.061) = -19.51 | 9.416619e-85 | -0.0429454 | -0.1476349 |
| Jul 2020 | Aug 2020 | t(735437.134) = -8.703 | 3.249563e-18 | -0.0200807 | -0.0705978 |
| Aug 2020 | Sept 2020 | t(708061.753) = -15.841 | 1.664891e-56 | -0.0369784 | -0.1319357 |
| Sept 2020 | Oct 2020 | t(577131.642) = 4.549 | 5.387777e-06 | 0.0117776 | 0.0423862 |
| Oct 2020 | Nov 2020 | t(553503.66) = -0.713 | 4.757907e-01 | -0.0019137 | -0.0068597 |
| Nov 2020 | Dec 2020 | t(542084.321) = -8.145 | 3.805390e-16 | -0.0220352 | -0.0794499 |
| Dec 2020 | Jan 2021 | t(526330.948) = 4.295 | 1.748367e-05 | 0.0118372 | 0.0427495 |
| Jan 2021 | Feb 2021 | t(399725.832) = -13.695 | 1.105459e-42 | -0.0414139 | -0.1506127 |
| Feb 2021 | Mar 2021 | t(719466.558) = -6.242 | 4.321693e-10 | -0.0147050 | -0.0552501 |

**Table S17**

*Welch’s t-tests between each month after the pandemic and the following month (March 2020-March 2021) for Collective-focused language in the Reddit dataset*

| Time period | | Collective Focused | | | | |
| --- | --- | --- | --- | --- | --- | --- |
| T1 | T2 | t-test | p-value | Cohen’s d | Mean diff | |
| Mar 2020 | Apr 2020 | t(719044.55) = -2.893 | 3.816118e-03 | -0.0068165 | -0.0114739 |  |
| Apr 2020 | May 2020 | t(724927.158) = 5.988 | 2.121644e-09 | 0.0140590 | 0.0233552 |  |
| May 2020 | Jun 2020 | t(726655.637) = 22.3 | 4.050360e-110 | 0.0489912 | 0.0770759 |  |
| Jun 2020 | Jul 2020 | t(722102.603) = -10.205 | 1.886576e-24 | -0.0224564 | -0.0347756 |  |
| Jul 2020 | Aug 2020 | t(721678.111) = 16.498 | 3.911887e-61 | 0.0382395 | 0.0588643 |  |
| Aug 2020 | Sept 2020 | t(715575.476) = 3.167 | 1.541923e-03 | 0.0073727 | 0.0111161 |  |
| Sept 2020 | Oct 2020 | t(579113.531) = 4.26 | 2.045432e-05 | 0.0110183 | 0.0164605 |  |
| Oct 2020 | Nov 2020 | t(555947.542) = -17.607 | 2.260916e-69 | -0.0471550 | -0.0721502 |  |
| Nov 2020 | Dec 2020 | t(545689.005) = 9.494 | 2.235655e-21 | 0.0256381 | 0.0398946 |  |
| Dec 2020 | Jan 2021 | t(526143.664) = 3.517 | 4.371306e-04 | 0.0096927 | 0.0147652 |  |
| Jan 2021 | Feb 2021 | t(396404.001) = -6.802 | 1.033354e-11 | -0.0206140 | -0.0317033 |  |
| Feb 2021 | Mar 2021 | t(402209.5) = -1.355 | 1.755325e-01 | -0.0042230 | -0.0066612 |  |

**Table S18**

*T-tests between each month period and time zero (March 2019) for Analytic Thinking in the Reddit Dataset.*

| Time period | | Analytic Thinking | | | |
| --- | --- | --- | --- | --- | --- |
| T1 | T2 | t-test | p-value | Cohen’s d | Mean diff |
| Time Zero | April 2019 | t(394205.879) = -3.321 | 8.982041e-04 | -0.0105591 | -0.3161002 |
| Time Zero | May 2019 | t(401566.954) = 2.061 | 3.934350e-02 | 0.0064997 | 0.1949149 |
| Time Zero | June 2019 | t(418869.739) = 0.978 | 3.278377e-01 | 0.0030212 | 0.0905745 |
| Time Zero | July 2019 | t(410826.387) = 1.609 | 1.075820e-01 | 0.0050211 | 0.1506369 |
| Time Zero | August 2019 | t(416801.35) = 2.726 | 6.406147e-03 | 0.0084420 | 0.2533514 |
| Time Zero | September 2019 | t(397961.828) = -0.096 | 9.235344e-01 | -0.0003040 | -0.0091069 |
| Time Zero | October 2019 | t(399631.325) = 0.449 | 6.536602e-01 | 0.0014184 | 0.0424761 |
| Time Zero | November 2019 | t(394848.475) = -0.157 | 8.753850e-01 | -0.0004983 | -0.0149292 |
| Time Zero | December 2019 | t(364579.671) = 2.234 | 2.551241e-02 | 0.0073115 | 0.2191623 |
| Time Zero | January 2020 | t(387976.614) = -1.832 | 6.696804e-02 | -0.0058630 | -0.1754983 |
| Time Zero | February 2020 | t(398543.628) = 0.591 | 5.546901e-01 | 0.0018698 | 0.0559240 |
| Time Zero | March 2020 | t(425114.281) = 45.136 | 0.000000e+00 | 0.1258752 | 3.7463428 |
| Time Zero | April 2020 | t(416611.441) = 52.729 | 0.000000e+00 | 0.1459023 | 4.3236539 |
| Time Zero | May 2020 | t(421512.954) = 42.981 | 0.000000e+00 | 0.1198246 | 3.5511246 |
| Time Zero | June 2020 | t(369085.846) = 40.073 | 0.000000e+00 | 0.1047020 | 3.1147802 |
| Time Zero | July 2020 | t(424702.732) = 33.559 | 1.384531e-246 | 0.0940160 | 2.7876053 |
| Time Zero | August 2020 | t(403823.491) = 26.993 | 2.457376e-160 | 0.0733743 | 2.1762151 |
| Time Zero | September 2020 | t(428739.754) = 26.67 | 1.413690e-156 | 0.0750717 | 2.2306115 |
| Time Zero | October 2020 | t(438114.495) = 23.297 | 5.635469e-120 | 0.0684534 | 2.0363525 |
| Time Zero | November 2020 | t(437995.935) = 28.52 | 9.642662e-179 | 0.0826318 | 2.4582553 |
| Time Zero | December 2020 | t(437622.883) = 26.287 | 3.595861e-152 | 0.0776984 | 2.3130053 |
| Time Zero | January 2021 | t(438568.212) = 28.862 | 5.336802e-183 | 0.0849609 | 2.5317545 |
| Time Zero | February 2021 | t(391627.891) = 20.038 | 2.863933e-89 | 0.0639021 | 1.9040811 |
| Time Zero | March 2021 | t(425452.307) = 23.15 | 1.702341e-118 | 0.0706776 | 2.1019096 |

**Table S19**

*T-tests between each month period and time zero (March 2019) for Cognitive Processing in the Reddit Dataset.*

| Time Period | | Cognitive Processing | | | |
| --- | --- | --- | --- | --- | --- |
| T1 | T2 | t-test | p-value | Cohen’s d | Mean diff |
| Time Zero | April 2019 | t(394241.506) = 2.949 | 3.188070e-03 | 0.0093772 | -0.0405649 |
| Time Zero | May 2019 | t(401623.632) = -0.304 | 7.612606e-01 | -0.0009583 | -0.1065350 |
| Time Zero | June 2019 | t(418869.429) = 2.18 | 2.924768e-02 | 0.0067316 | -0.2035422 |
| Time Zero | July 2019 | t(410825.972) = 6.219 | 5.002957e-10 | 0.0194057 | 0.0948325 |
| Time Zero | August 2019 | t(416752.266) = 1.931 | 5.350156e-02 | 0.0059792 | 0.1087069 |
| Time Zero | September 2019 | t(398101.838) = 1.206 | 2.276938e-01 | 0.0038200 | 0.0975957 |
| Time Zero | October 2019 | t(399768.577) = 0.639 | 5.231121e-01 | 0.0020184 | -0.0496766 |
| Time Zero | November 2019 | t(395016.036) = 0.07 | 9.444350e-01 | 0.0002214 | 0.0360969 |
| Time Zero | December 2019 | t(365029.166) = 1.534 | 1.249484e-01 | 0.0050210 | 0.0824347 |
| Time Zero | January 2020 | t(388283.705) = 3.113 | 1.849613e-03 | 0.0099623 | -0.0485719 |
| Time Zero | February 2020 | t(398678.677) = 0.613 | 5.398103e-01 | 0.0019404 | 0.2424028 |
| Time Zero | March 2020 | t(426863.949) = -8.516 | 1.650160e-17 | -0.0237191 | -0.1309687 |
| Time Zero | April 2020 | t(416107.067) = -11.03 | 2.767225e-28 | -0.0305324 | -0.0405649 |
| Time Zero | May 2020 | t(421398.28) = -17.167 | 4.956177e-66 | -0.0478633 | -0.1065350 |
| Time Zero | June 2020 | t(374276.387) = -30.751 | 2.151792e-207 | -0.0797938 | -0.2035422 |
| Time Zero | July 2020 | t(425515.475) = -23.37 | 1.035383e-120 | -0.0654302 | 0.0948325 |
| Time Zero | August 2020 | t(404992.282) = -17.572 | 4.258290e-69 | -0.0477136 | 0.1087069 |
| Time Zero | September 2020 | t(429816.868) = -11.311 | 1.174975e-29 | -0.0318128 | 0.0975957 |
| Time Zero | October 2020 | t(439242.984) = -13.55 | 8.091497e-42 | -0.0397869 | -0.0496766 |
| Time Zero | November 2020 | t(439204.601) = -11.727 | 9.358448e-32 | -0.0339516 | 0.0360969 |
| Time Zero | December 2020 | t(437819.005) = -6.984 | 2.872986e-12 | -0.0206411 | 0.0824347 |
| Time Zero | January 2021 | t(440185.047) = -9.65 | 4.939188e-22 | -0.0283794 | -0.0485719 |
| Time Zero | February 2021 | t(391849.384) = 3.383 | 7.174877e-04 | 0.0107863 | 0.2424028 |
| Time Zero | March 2021 | t(425354.726) = -3.431 | 6.010849e-04 | -0.0104757 | -0.1309687 |

**Table S20**

*T-tests between each month period and time zero (March 2019) for Self-focused usage in the Reddit Dataset.*

| Time Period | Self-focused | | | | |
| --- | --- | --- | --- | --- | --- |
| T1 | T2 | t-test | p-value | Cohen’s d | Mean diff |
| Time Zero | April 2019 | t(394050.217) = 0.247 | 8.051937e-01 | 0.0007843 | 0.0028479 |
| Time Zero | May 2019 | t(401789.761) = 3.951 | 7.796754e-05 | 0.0124595 | 0.0450844 |
| Time Zero | June 2019 | t(418756.304) = 3.266 | 1.089790e-03 | 0.0100859 | 0.0365710 |
| Time Zero | July 2019 | t(410828.774) = 0.094 | 9.250333e-01 | 0.0002936 | 0.0010679 |
| Time Zero | August 2019 | t(416519.513) = 2.02 | 4.338802e-02 | 0.0062559 | 0.0226354 |
| Time Zero | September 2019 | t(398274.751) = 7.564 | 3.904266e-14 | 0.0239511 | 0.0865788 |
| Time Zero | October 2019 | t(399775.794) = 8.234 | 1.816095e-16 | 0.0260270 | 0.0942151 |
| Time Zero | November 2019 | t(395200.054) = 8.699 | 3.361193e-18 | 0.0276351 | 0.0999390 |
| Time Zero | December 2019 | t(363770.737) = 1.79 | 7.338227e-02 | 0.0058642 | 0.0213530 |
| Time Zero | January 2020 | t(387850.875) = 1.732 | 8.330740e-02 | 0.0055431 | 0.0201141 |
| Time Zero | February 2020 | t(398452.329) = 3.507 | 4.525351e-04 | 0.0111021 | 0.0402654 |
| Time Zero | March 2020 | t(438779.235) = -3.594 | 3.260138e-04 | -0.0099195 | -0.0366621 |
| Time Zero | April 2020 | t(437353.73) = -9.063 | 1.269305e-19 | -0.0246664 | -0.0919122 |
| Time Zero | May 2020 | t(430169.618) = 2.164 | 3.044244e-02 | 0.0059939 | 0.0218554 |
| Time Zero | June 2020 | t(351730.066) = 43.045 | 0.000000e+00 | 0.1152680 | 0.3993584 |
| Time Zero | July 2020 | t(417795.8) = 25.203 | 4.690801e-140 | 0.0709724 | 0.2517235 |
| Time Zero | August 2020 | t(399584.253) = 18.62 | 2.383862e-77 | 0.0508161 | 0.1811257 |
| Time Zero | September 2020 | t(431709.729) = 4.839 | 1.302574e-06 | 0.0135932 | 0.0491901 |
| Time Zero | October 2020 | t(438061.655) = 8.651 | 5.143158e-18 | 0.0254186 | 0.0915763 |
| Time Zero | November 2020 | t(438129.611) = 8.113 | 4.956980e-16 | 0.0235036 | 0.0847165 |
| Time Zero | December 2020 | t(439847.653) = 0.492 | 6.228609e-01 | 0.0014518 | 0.0052666 |
| Time Zero | January 2021 | t(438735.105) = 4.518 | 6.252788e-06 | 0.0132975 | 0.0480162 |
| Time Zero | February 2021 | t(389713.375) = -8.78 | 1.641632e-18 | -0.0280336 | -0.1025966 |
| Time Zero | March 2021 | t(428179.377) = -12.161 | 5.084234e-34 | -0.0370654 | -0.1359228 |

**Table S21**

*T-tests between each month period and time zero (March 2019) for Collective-focused usage in the Reddit Dataset.*

| Time Period | Collective-focused | | | | |
| --- | --- | --- | --- | --- | --- |
| T1 | T2 | t-test | p-value | Cohen’s d | Mean diff |
| Time Zero | April 2019 | t(393925.943) = 0.298 | 7.657404e-01 | 0.0009476 | 0.0013065 |
| Time Zero | May 2019 | t(401410.417) = -0.594 | 5.526561e-01 | -0.0018731 | -0.0025867 |
| Time Zero | June 2019 | t(419447.344) = -4.161 | 3.165713e-05 | -0.0128430 | -0.0178597 |
| Time Zero | July 2019 | t(410814.606) = -3.169 | 1.528991e-03 | -0.0098885 | -0.0136881 |
| Time Zero | August 2019 | t(416623.48) = 0.897 | 3.695186e-01 | 0.0027791 | 0.0038201 |
| Time Zero | September 2019 | t(397547.774) = -3.185 | 1.447893e-03 | -0.0100891 | -0.0139486 |
| Time Zero | October 2019 | t(399072.206) = -3.241 | 1.189340e-03 | -0.0102502 | -0.0141963 |
| Time Zero | November 2019 | t(395157.039) = -0.462 | 6.442111e-01 | -0.0014672 | -0.0020135 |
| Time Zero | December 2019 | t(363724.878) = -1.767 | 7.722311e-02 | -0.0057877 | -0.0079951 |
| Time Zero | January 2020 | t(385779.276) = -4.601 | 4.211925e-06 | -0.0147434 | -0.0205430 |
| Time Zero | February 2020 | t(398553.436) = -0.347 | 7.286932e-01 | -0.0010979 | -0.0015084 |
| Time Zero | March 2020 | t(496557.144) = -56.496 | 0.000000e+00 | -0.1492494 | -0.2349009 |
| Time Zero | April 2020 | t(497164.532) = -59.859 | 0.000000e+00 | -0.1555442 | -0.2463747 |
| Time Zero | May 2020 | t(487395.221) = -54.461 | 0.000000e+00 | -0.1444294 | -0.2230195 |
| Time Zero | June 2020 | t(411534.81) = -39.465 | 0.000000e+00 | -0.0979308 | -0.1459436 |
| Time Zero | July 2020 | t(476130.396) = -44.578 | 0.000000e+00 | -0.1202096 | -0.1807192 |
| Time Zero | August 2020 | t(444598.344) = -31.68 | 5.094236e-220 | -0.0830715 | -0.1218550 |
| Time Zero | September 2020 | t(462864.816) = -27.662 | 2.741880e-168 | -0.0759699 | -0.1107388 |
| Time Zero | October 2020 | t(455393.708) = -22.599 | 5.067485e-113 | -0.0656498 | -0.0942783 |
| Time Zero | November 2020 | t(472512.6) = -39.352 | 0.000000e+00 | -0.1112486 | -0.1664285 |
| Time Zero | December 2020 | t(457668.495) = -29.603 | 2.066164e-192 | -0.0862507 | -0.1265339 |
| Time Zero | January 2021 | t(457939.769) = -26.449 | 4.900695e-154 | -0.0768219 | -0.1117687 |
| Time Zero | February 2021 | t(376311.891) = -30.338 | 6.371532e-202 | -0.0973275 | -0.1434720 |
| Time Zero | March 2021 | t(430068.141) = -33.311 | 5.520932e-243 | -0.1009471 | -0.1501332 |

**Table S22**

*Simple Pearson Correlations between Reddit and CEO data on variables of interest*

| Variable | r | P value |
| --- | --- | --- |
| Analytic Thinking | 0.60 | < 0.001 |
| Cognitive Processing | 0.57 | 0.002 |
| Self-focused | -0.12 | 0.57 |
| Collective-focused | -0.68 | < 0.001 |

**VI. Post-Hoc Statistical Tests**

**Table S23.**

*T-tests between each quarterly period and time zero (Q1 2020) for analytic thinking*

| Time period | | Analytic Thinking | | | |
| --- | --- | --- | --- | --- | --- |
| T1 | T2 | t-test | p-value | Cohen’s D | Mean diff |
| Time Zero | Q1 2010 | t(1383) = -7.7895 | 1.312e-14 | -0.1851029 | -2.5595130 |
| Time Zero | Q2 2010 | t(1752.3) = -5.7883 | 8.408e-09 | -0.1339318 | -1.8237112 |
| Time Zero | Q3 2010 | t(1398.2) = -3.9581 | 7.936e-05 | -0.222725 | -3.1031328 |
| Time Zero | Q4 2010 | t(1316.8) = -2.8288 | 0.004744 | -0.1475299 | -2.0355221 |
| Time Zero | Q1 2011 | t(1104.2) = -4.4475 | 9.564e-06 | -0.06572546 | -0.9202096 |
| Time Zero | Q2 2011 | t(1326.8) = -3.1111 | 0.001904 | -0.03417595 | -0.4735812 |
| Time Zero | Q3 2011 | t(1700.9) = -1.4822 | 0.1385 | -0.1166355 | -1.5973706 |
| Time Zero | Q4 2011 | t(1728.6) = -0.77436 | 0.4388 | -0.07966127 | -1.0953330 |
| Time Zero | Q1 2012 | t(1638.9) = -2.6067 | 0.009224 | -0.09838845 | -1.3665653 |
| Time Zero | Q2 2013 | t(1889.5) = -1.8502 | 0.06445 | -0.03778807 | -0.5164610 |
| Time Zero | Q3 2013 | t(2364.2) = -2.4441 | 0.01459 | -0.09185766 | -1.2581615 |
| Time Zero | Q4 2013 | t(2496.6) = -0.95437 | 0.34 | -0.03645933 | -0.5232129 |
| Time Zero | Q1 2014 | t(2555.6) = -2.34 | 0.01936 | -0.0492566 | -0.6991442 |
| Time Zero | Q2 2014 | t(3156.4) = -1.0725 | 0.2836 | -0.01517573 | -0.2083640 |
| Time Zero | Q3 2014 | t(3065.3) = -1.3838 | 0.1665 | -0.1048534 | -1.4530301 |
| Time Zero | Q4 2014 | t(3004.1) = -0.42289 | 0.6724 | -0.09567371 | -1.3405792 |
| Time Zero | Q1 2015 | t(2715.2) = -2.7395 | 0.006194 | -0.0797918 | -1.1050628 |
| Time Zero | Q2 2015 | t(3089.7) = -2.7727 | 0.005593 | -0.05884322 | -0.8215474 |
| Time Zero | Q3 2015 | t(3047.3) = -2.2597 | 0.02391 | -0.1173865 | -1.5740651 |
| Time Zero | Q4 2015 | t(3074.4) = -1.6814 | 0.09279 | -0.09435632 | -1.3251540 |
| Time Zero | Q1 2016 | t(2762.8) = -3.0863 | 0.002047 | -0.06937799 | -0.9586343 |
| Time Zero | Q2 2016 | t(3018.9) = -2.8576 | 0.004298 | -0.00764985 | -0.1047645 |
| Time Zero | Q3 2016 | t(3050.2) = -1.9896 | 0.04672 | -0.02956516 | -0.4018354 |
| Time Zero | Q4 2016 | t(3023.2) = -0.21975 | 0.8261 | -0.0517852 | -0.7327220 |

**Decade Analyses**

**Table S24**

*T-tests between each quarterly period and time zero (Q1 2020) for cognitive processing language*

| Time period | | Cognitive Processing | | | |
| --- | --- | --- | --- | --- | --- |
| T1 | T2 | t-test | p-value | Cohen’s D | Mean diff |
| Time Zero | Q1 2010 | t(1570) = -3.6648 | 0.0002557 | -0.1644938 | -0.35852957 |
| Time Zero | Q2 2010 | t(1448.8) = -2.5542 | 0.01074 | -0.1166419 | -0.25350215 |
| Time Zero | Q3 2010 | t(1268.9) = -1.7089 | 0.08771 | -0.08071814 | -0.17664648 |
| Time Zero | Q4 2010 | t(1432.2) = -3.1437 | 0.001703 | -0.1449077 | -0.32039837 |
| Time Zero | Q1 2011 | t(1903.6) = -4.8415 | 1.393e-06 | -0.2075203 | -0.45266995 |
| Time Zero | Q2 2011 | t(1856) = -4.4917 | 7.501e-06 | -0.1940441 | -0.42817462 |
| Time Zero | Q3 2011 | t(1671.9) = -2.555 | 0.01071 | -0.1136 | -0.25461808 |
| Time Zero | Q4 2011 | t(2041.8) = -2.5502 | 0.01084 | -0.1073236 | -0.23308017 |
| Time Zero | Q1 2012 | t(2449) = -3.0489 | 0.002321 | -0.1217299 | -0.26827971 |
| Time Zero | Q2 2013 | t(2516) = -3.4394 | 0.0005925 | -0.1359451 | -0.30345757 |
| Time Zero | Q3 2013 | t(2590.9) = -2.7545 | 0.00592 | -0.1077753 | -0.23728250 |
| Time Zero | Q4 2013 | t(2917.9) = -3.8344 | 0.0001285 | -0.1335815 | -0.29365826 |
| Time Zero | Q1 2014 | t(3005.4) = -4.1395 | 3.576e-05 | -0.1483548 | -0.33622255 |
| Time Zero | Q2 2014 | t(2878.6) = -3.3617 | 0.0007848 | -0.1219518 | -0.26262232 |
| Time Zero | Q3 2014 | t(2729.5) = -1.3939 | 0.1635 | -0.05332292 | -0.11908550 |
| Time Zero | Q4 2014 | t(2937.2) = -2.929 | 0.003427 | -0.1026109 | -0.22612302 |
| Time Zero | Q1 2015 | t(2930.8) = -2.0681 | 0.03872 | -0.07383083 | -0.16171720 |
| Time Zero | Q2 2015 | t(2974.6) = -1.2311 | 0.2184 | -0.04350875 | -0.09674229 |
| Time Zero | Q3 2015 | t(2750) = -1.0928 | 0.2746 | -0.04156669 | -0.08945608 |
| Time Zero | Q4 2015 | t(2879.2) = -2.6399 | 0.008339 | -0.08858094 | -0.19792298 |
| Time Zero | Q1 2016 | t(2826) = -1.2087 | 0.2269 | -0.04303704 | -0.09159200 |
| Time Zero | Q2 2016 | t(2872.3) = -1.9393 | 0.05257 | -0.06848693 | -0.14795516 |
| Time Zero | Q3 2016 | t(2733.2) = -1.4796 | 0.1391 | -0.05639169 | -0.12031069 |
| Time Zero | Q4 2016 | t(2747.2) = -2.8914 | 0.003866 | -0.09645616 | -0.21175817 |

**Table S25**

*T-tests between each quarterly period and time zero (Q1 2020) for Collective-focus language.*

| Time period | | Collective-focus | | | |
| --- | --- | --- | --- | --- | --- |
| T1 | T2 | t-test | p-value | Cohen’s D | Mean diff |
| Time Zero | Q1 2010 | t(1470.6) = 6.6342 | 4.572e-11 | 0.3047496 | 0.4359992 |
| Time Zero | Q2 2010 | t(1340.3) = -4.8939 | 1.108e-06 | 0.2301494 | 0.3290490 |
| Time Zero | Q3 2010 | t(1186.1) = 4.1053 | 4.315e-05 | 0.1993649 | 0.2855660 |
| Time Zero | Q4 2010 | t(1346.5) = 4.1082 | 4.229e-05 | 0.1937381 | 0.2808382 |
| Time Zero | Q1 2011 | t(1834.7) = 4.3293 | 1.576e-05 | 0.1877219 | 0.2669796 |
| Time Zero | Q2 2011 | t(1781.7) = 5.4225 | 6.681e-08 | 0.2371892 | 0.3419809 |
| Time Zero | Q3 2011 | t(1634.6) = 4.157 | 3.392e-05 | 0.1861524 | 0.2698555 |
| Time Zero | Q4 2011 | t(1908) = 5.2693 | 1.524e-07 | 0.2262647 | 0.3270471 |
| Time Zero | Q1 2012 | t(2378.7) = 4.9981 | 6.211e-07 | 0.200949 | 0.2931861 |
| Time Zero | Q2 2013 | t(2500.8) = 4.7696 | 1.952e-06 | 0.1887823 | 0.2721081 |
| Time Zero | Q3 2013 | t(2535.5) = 4.0633 | 4.986e-05 | 0.1597291 | 0.2342147 |
| Time Zero | Q4 2013 | t(3090) = 4.7183 | 2.484e-06 | 0.1615416 | 0.2395374 |
| Time Zero | Q1 2014 | t(3056) = 5.4596 | 5.155e-08 | 0.1945666 | 0.2901721 |
| Time Zero | Q2 2014 | t(3020.1) = 3.6757 | 0.0002413 | 0.131684 | 0.1927559 |
| Time Zero | Q3 2014 | t(2708.5) = 3.9876 | 6.851e-05 | 0.1526475 | 0.2253730 |
| Time Zero | Q4 2014 | t(3036.5) = 5.82 | 6.499e-09 | 0.2019221 | 0.2937306 |
| Time Zero | Q1 2015 | t(3068.5) = 4.2671 | 2.04e-05 | 0.1503406 | 0.2221707 |
| Time Zero | Q2 2015 | t(3121.1) = 3.0993 | 0.001957 | 0.107909 | 0.1625054 |
| Time Zero | Q3 2015 | t(2762.4) = 4.3095 | 1.694e-05 | 0.1639076 | 0.2350808 |
| Time Zero | Q4 2015 | t(3050.3) = 5.0093 | 5.775e-07 | 0.1648146 | 0.2468167 |
| Time Zero | Q1 2016 | t(3014.9) = 4.5392 | 5.867e-06 | 0.1588378 | 0.2291996 |
| Time Zero | Q2 2016 | t(3073.9) = 3.8426 | 0.0001242 | 0.1330847 | 0.1960384 |
| Time Zero | Q3 2016 | t(2746.5) = 3.8237 | 0.0001344 | 0.1457918 | 0.2103506 |
| Time Zero | Q4 2016 | t(2875.8) = 4.5861 | 4.708e-06 | 0.1504828 | 0.2188209 |

**Table S26**

*T-tests between each quarterly period and time zero (Q1 2020) for Self-focus language.*

| Time period | | Self-focus | | | |
| --- | --- | --- | --- | --- | --- |
| T1 | T2 | t-test | p-value | Cohen’s D | Mean diff |
| Time Zero | Q1 2010 | t(1543.5) = 1.2878 | 0.198 | -0.05815116 | -0.050134242 |
| Time Zero | Q2 2010 | t(1384.9) = -0.10864 | 0.9135 | -0.005046179 | -0.004378159 |
| Time Zero | Q3 2010 | t(1243.2) = -1.623 | 0.1048 | -0.07729913 | -0.066922474 |
| Time Zero | Q4 2010 | t(1407) = -0.99487 | 0.32 | -0.04615945 | -0.040383399 |
| Time Zero | Q1 2011 | t(1807.3) = -2.5069 | 0.01227 | -0.1091999 | -0.096135757 |
| Time Zero | Q2 2011 | t(1839.8) = -3.3245 | 0.0009033 | -0.1440105 | -0.125450179 |
| Time Zero | Q3 2011 | t(1676.7) = -2.5305 | 0.01148 | -0.1124059 | -0.098887315 |
| Time Zero | Q4 2011 | t(1968.6) = -2.0998 | 0.03587 | -0.0893624 | -0.077916545 |
| Time Zero | Q1 2012 | t(2437.1) = -1.2366 | 0.2164 | -0.04943775 | -0.043091943 |
| Time Zero | Q2 2013 | t(2522.1) = -1.3458 | 0.1785 | -0.05316316 | -0.046460674 |
| Time Zero | Q3 2013 | t(2584.1) = -1.1618 | 0.2454 | -0.04549139 | -0.039612265 |
| Time Zero | Q4 2013 | t(2968.4) = -1.4194 | 0.1559 | -0.04920324 | -0.043144470 |
| Time Zero | Q1 2014 | t(3026.8) = -2.3974 | 0.01657 | -0.08572923 | -0.077202072 |
| Time Zero | Q2 2014 | t(2993.6) = -2.0674 | 0.03879 | -0.07426657 | -0.065868147 |
| Time Zero | Q3 2014 | t(2732) = -0.71692 | 0.4735 | -0.02741784 | -0.023714009 |
| Time Zero | Q4 2014 | t(2965.1) = -0.10205 | 0.9187 | -0.00356539 | -0.003114283 |
| Time Zero | Q1 2015 | t(3004) = 0.59074 | 0.5547 | 0.02094931 | 0.018513587 |
| Time Zero | Q2 2015 | t(2941.8) = -0.2985 | 0.7653 | -0.01058133 | -0.009147220 |
| Time Zero | Q3 2015 | t(2746.3) = 1.0658 | 0.2866 | 0.0405406 | 0.034143145 |
| Time Zero | Q4 2015 | t(3052.2) = 1.0236 | 0.3061 | 0.03367177 | 0.030988407 |
| Time Zero | Q1 2016 | t(2945) = 1.1994 | 0.2305 | 0.04225072 | 0.036607623 |
| Time Zero | Q2 2016 | t(2915) = 0.72184 | 0.4704 | 0.02539163 | 0.021830239 |
| Time Zero | Q3 2016 | t(2749.5) = -0.70012 | 0.4839 | -0.02669258 | -0.023391960 |
| Time Zero | Q4 2016 | t(2859.9) = 1.3196 | 0.1871 | 0.04338585 | 0.038585908 |

**Supplementary References**

1. Chung CK, Pennebaker JW. 2008. Revealing dimensions of thinking in open-ended self-descriptions: An automated meaning extraction method for natural language. Journal of research in personality. 42(1):96–132.
2. Markowitz DM. 2021. The meaning extraction method: An approach to evaluate content patterns from large-scale language data. Frontiers in Communication. 6:13.
3. Vine V, Boyd RL, Pennebaker JW. 2020. Natural emotion vocabularies as windows on distress and well-being. Nature communications. 11(1):1–9.
4. Slatcher RB, Pennebaker JW. 2006. How do I love thee? Let me count the words: The social effects of expressive writing. Psychological Science. 17(8):660–664.
5. Seraj S, Blackburn KG, Pennebaker JW. 2021. Language left behind on social media exposes the emotional and cognitive costs of a romantic breakup. Proc Natl Acad Sci USA. 118(7):e2017154118. doi:[10.1073/pnas.2017154118](https://doi.org/10.1073/pnas.2017154118).
6. Ashokkumar A, Pennebaker JW. 2021. Social media conversations reveal large psychological shifts caused by COVID-19’s onset across US cities. Science advances. 7(39):eabg7843.
7. Pennebaker JW, Boyd RL, Booth RJ, Ashokkumar A, Francis ME. Linguistic Inquiry and Word Count: LIWC-22. 2022. www.liwc.app
8. Chung C, Pennebaker JW. 2007. The psychological functions of function words. Social communication. 1:343–359.
9. Tausczik YR, Pennebaker JW. 2010. The Psychological Meaning of Words: LIWC and Computerized Text Analysis Methods. Journal of Language and Social Psychology. 29(1):24–54. doi:[10.1177/0261927X09351676](https://doi.org/10.1177/0261927X09351676).
10. Kacewicz E, Pennebaker JW, Davis M, Jeon M, Graesser AC. 2014. Pronoun Use Reflects Standings in Social Hierarchies. Journal of Language and Social Psychology. 33(2):125–143. doi:[10.1177/0261927X13502654](https://doi.org/10.1177/0261927X13502654).
11. Pennebaker JW, Stone LD. 2003. Words of wisdom: language use over the life span. Journal of personality and social psychology. 85(2):291.
12. Pezzuti T, Leonhardt JM, Warren C. 2021. Certainty in language increases consumer engagement on social media. Journal of Interactive Marketing. 53:32–46.
13. Pennebaker JW, Mehl MR, Niederhoffer KG. 2003. Psychological Aspects of Natural Language Use: Our Words, Our Selves. Annu Rev Psychol. 54(1):547–577. doi:[10.1146/annurev.psych.54.101601.145041](https://doi.org/10.1146/annurev.psych.54.101601.145041).
