# Supplementary material for: The psychological impacts of the COVID-19 pandemic on business leadership
Source: PLoS One. 2023 Oct 11;18(10):e0290621. doi: 10.1371/journal.pone.0290621 (PMC10566739; doi:10.1371/journal.pone.0290621)
Supplement: Attachment — Submitted filename: Response Letter.docx [file pone.0290621.s002.docx]

Dear Editor,

We greatly appreciate the thoughtful comments on our manuscript titled, “**The Psychological Impacts of the COVID-19 Pandemic on Business Leadership**''. As requested by the reviewers, we streamlined the paper and framed the language change of CEOs relative to that of a more general population. We carefully considered each of the points raised and believe that the resulting major overhaul of the manuscript significantly strengthened and improved the paper overall. The revised manuscript is attached along with this detailed letter that contains responses to each concern raised. In particular, in response to Reviewer 1’s suggestions, we provide a comparison of CEOs’ language change relative to the general public using Reddit data. We also made substantial changes and clarifications in response to Reviewer 2’s suggestions. Below, we detail point-by-point how we have addressed each reviewer suggestion, with the original reviewer comments in blue, our response indented, and selected quotes from the revised manuscript in italics.

Thank you for all the suggestions and support. We look forward to hearing back from you.

Steven Mesquiti and Sarah Seraj

----

**Editor Prompts**:

a) If there are ethical or legal restrictions on sharing a de-identified data set, please explain them in detail (e.g., data contain potentially sensitive information, data are owned by a third-party organization, etc.) and who has imposed them (e.g., an ethics committee). Please also provide contact information for a data access committee, ethics committee, or other institutional body to which data requests may be sent.

There are no ethical or legal restrictions on sharing the de-identified dataset.

b) If there are no restrictions, please upload the minimal anonymized data set necessary to replicate your study findings as either Supporting Information files or to a stable, public repository and provide us with the relevant URLs, DOIs, or accession numbers. For a list of acceptable repositories, please see http://journals.plos.org/plosone/s/data-availability#loc-recommended-repositories.

The anonymized datasets are available at the following link: : https://osf.io/sybea/?view_only=5812674bd2ba4f618318bb94725ba443 for the anonymized datasets necessary to replicate our study.

**Reviewer #1**:

The manuscript provides an interesting aspect of the pandemic impacts and offers unique insights on how economic leaders’ psychology shifted during the pandemic. The analysis is done based on the sound theoretical framework. My major concern at the sane time is the focus on CEOs. While this is an interesting angle and motivated well in the introduction, the findings should be able to highlight some unique aspects of CEOs. That being said, the manuscript should provide some comparisons to general public or other cohorts. Else, the focus on CEOs cannot be justified. Any perspective on this should be addressed in the discussion section using prior literature or in the limitation section.

Thank you for your comments. We agree that contextualizing our findings with that of a more general population is rather important. As a result, we have included some comparisons to the general public by analyzing and adding Reddit data to our graphs (10,738,506 comments from 834,172 users in 22 US city subreddits in the period from March 2019 to March 2021) . Please see Methods section for more information on data collection). Analyses with the additional data is now incorporated into the Results and Discussion as well as Figures 1 and 2 (see below). We have also highlighted some of the unique aspects of CEOs in comparison with the general public.

**Fig 1. CEOs' Language Change.** Change in language patterns of Reddit users and CEOs before and after the pandemic. The red line represents Reddit post history in the displayed time range (*N* = 10,738,506 posts). The blue line refers to all CEO transcripts in the displayed time range (*N* = 19,536 calls).Vertical Line Represents the onset of the pandemic. Each point is the average score across CEOS (*N* = 3,044) Reddit users (*N* = 834,172) at that time block (1 month intervals). The horizontal shaded areas are 95% CIs for each datapoint, vertical sharing represent virus surges. The self-focused language is measured using I-words and collective-focused using we-words.

**Fig 2. CEOs' Language Change over the Decade.** Change in language patterns of Reddit users and CEOs before and after the pandemic. The red line represents Reddit post history in the displayed time range (*N =* 33.7 million posts). The blue line refers to all CEO transcripts in the displayed time range (*N* = 79,725 transcripts). The vertical Line Represents the onset of the pandemic. Each point is the average score across CEOs (*N* = 4,707 CEO) and Redditors (*N* = 1.37 million) at that time block (1 quarter intervals; 3 months). The horizontal shaded areas are 95% CIs for each datapoint, vertical sharing represent virus surges. The self-focused language is measured using I-words and collective-focused using we-words. Effect sizes presented are the average effect size at 5 and 10 years for the CEO dataset.

Further, some editing as well as restructuring seem necessary. Currently, the manuscript reads more like a short version of thesis or dissertation. The introduction should be shorter and more succinct. The first paragraph under Method is not a method. There should be another section for Data and Material (or alike).

- Thank you for your constructive comments, which have greatly strengthened the manuscript. We have restructured the paper and condensed some of the sections. As per your feedback, we also have a separate Data Section.

**Reviewer #2**:

Title: “The Effects of COVID-19 Pandemic on CEO Language Change” is more appropriate and accurate as a title than “The Psychological Impacts of the COVID-19 Pandemic on Corporate Leadership” Perhaps, authors could mix both titles.

We appreciate your insight here. We have switched the title to: “The Psychological Impacts of the COVID-19 Pandemic on Business Leadership” to better capture the nature of the paper.

Abstract: It would be useful to include a broad description of the conclusions in the abstract.

Thank you for the feedback. We have updated the abstract to add the big picture conclusions, which are reproduced below:
*The observed language changes reflect the unique effect of the pandemic on CEOs, which had some notable differences compared to the general population. This study sheds light on how the COVID-19 pandemic influenced business leaders' psychological states and decision-making strategies—processes that have a substantial impact on a company’s performance. The findings underscore the importance of language data in understanding large-scale societal events.*

Keywords. COVID, Pandemic and Leadership keywords are redundant with the title. Perhaps, authors could include another keywords.Perhaps “Linguistic Inquiry and Word Count” is more appropriate as a keyword than LIWC.

- We appreciate your feedback here. The updated keywords are now: *LIWC, COVID, Language, CEOs, Social Upheaval*. Since LIWC is widely used as an acronym for Linguistic Inquiry and Word Count, we still kept “LIWC” as a keyword in the spirit of parsimony.

Introduction

The introduction should include more aspects that describes the reasons why interest in the study Linguistic Inquiry and Word Count approach has increased (or the benefits of its increase) and what gaps this research intends to cover on Corporate Leadership analyses.

We appreciate your feedback. We now touch on how past social stage models of coping might mirror that of CEOs, as well as how CEOs might differ from lay people on page 5. Also on page 5, we provide empirical evidence that establishes some of the unique interpersonal qualities of CEOs, as well as how language analysis (specifically the analysis of function words) might allow us to test whether these unique qualities were present during the pandemic.

Methods

Authors follow a systematic approach. The raw sample extracted from Finnhub possessed 79,725 transcripts from 4,707 CEOs. Could authors analyze other sociodemographic variables (i.e. age, social class) or corporate characteristics (i.e. primary secondary or tertiary sector companies)? I suggest including these variables as covariates in the analyses.Please also create a first table presenting the characteristics of the sample.

Thank you very much for your clarifying question. Unfortunately, these variables are not readily available in this dataset due to it only possessing the names and not ethnicity nor gender. However, we provide some general information regarding the demographic qualities of CEOs on page 10:

”*Although it is difficult to obtain precise information about the demographics of individual CEOs, aggregated demographic information suggest that CEOs are largely upper middle class, white males, in middle to late adulthood [Larcker & Tayan, 2020]*...”

Authors state on page10 that “Extremely short texts of less than 25 words were omitted”. Short texts are common in open-sourced data on platforms. Why did the authors excluded them?

Thank you for raising this point. We have addressed this comment on page 10:

*Transcripts of less than 25 words were excluded from analyses, which is a common cutoff for bag-of-words text analysis approaches like the one our team used (N = 191 transcripts excluded).*

Essentially, Short transcripts would cause output to be excessively noisy, since LIWC’s output calculates the percentage of total words that pertain to different categories. Moreover, the 25 word cutoff only excludes a small percentage of texts in our dataset, usually ones that don’t have much information to begin with since they are quick replies to questions instead of a more detailed answer.

How many researchers are resolving the conflicts (i.e. when they prepared text samples for LIWC analysis)?

The earning calls transcripts are part of a well-maintained database through Finnhub, which aims to democratize financial data and make it more accessible to analysts. We did not have to do any additional cleaning of the text samples to process it through LIWC. As such, there weren’t any conflicts to resolve.

Authors state on page 9 that “Using the Finnhub API, all of the unscripted earnings calls for companies on the New York Stock Exchange between January of 2006 and March of 2021 were extracted. Nevertheless they stated on page 10 that “The raw sample extracted from Finnhub possessed 79,725 transcripts from 4,707 CEOs. From that, a sample of all companies in the database ranging from March 2019 to March 2021 was constructed, to acquire one year of data before and after lockdowns in the US.Have the analysed 2006-2021 data or from 2019?

Thank you for your clarifying question. We obtained all data from 2006-2021 from the Finnhub API. However, we only analyzed data from 2010-2021 due to there being a scarce number of observations before 2010. This point is now made more clear in the Methods section on page 10:

“*Using the Finnhub API, all unscripted earnings calls for companies on the New York Stock Exchange between January of 2006 and March of 2021 were extracted (N = 79,725 transcripts; N = 4,707 CEOs). Due to the low volume and temporal resolution of data before 2010, we excluded data from 2006-9 in our analyses.*

Results

Authors state on page 12 that “To analyze language shifts in CEOs, Welch’s paired t-tests were conducted to compare the established reference point of March 2019 against each month in the period of interest.”Welch’s t-test is generally applied when the there is a difference between the variations of two populations and also when their sample sizes are unequal. What criteria was used to select this statistical analysis?

We used Welch’s paired t-tests to account for an unequal number of calls in each month, as these calls can occur at any point in a fiscal quarter. We have added the following information to page 13: “

*Welch's paired t-tests were conducted to compare the established reference point of March 2019 against each month in the period of interest. We used Welch’s paired t-tests to account for an unequal number of calls in each month.”*

Discussion

Authors recognized some limitations on page 20. Nevertheless, Do the characteristics of the program used for the analysis allow it to capture the non-literal meaning of the expressions or the influence of the context on what is expressed? It would be useful to explain the limitations of the Linguistic Inquiry and Word Count 22 program.

Thank you for your clarifying question. We have added the following information to page 26:

“*While using LIWC allows for the exploration of psychological states via text analysis, it is not without its limitations. As a closed vocabulary approach, its internal dictionaries are not exhaustive. LIWC also does not take into account the context in which words are used [Schwartz et al., 2013] unlike large language models [LLM] such as Bidirectional Encoder Representations from Transformers [commonly referred to as BERT; Devlin et al., 2018] or Generative Pre-trained Transformer [the LLM developed by OpenAI that powers the chatbot ChatGPT; Brown et al., 2023]. While these LLMs provide more accurate results when it comes to context-specific settings, they are computationally intensive and harder to interpret compared to the LIWC method, which is more accessible.*”
